# PD30 How Is Sustainability Incorporated Into Healthcare Systems? A Progress Report Card For The EU5

**DOI:** 10.1017/S0266462324002939

**Published:** 2025-01-07

**Authors:** Laurie Marlow, Sarah Webb, Joanna Sulanowska

## Abstract

**Introduction:**

In 2020, the World Health Organization (WHO) released guidance for climate-resistant and environmentally sustainable healthcare facilities. Implementation of sustainability into healthcare policy is at varying stages, and its impact on technology appraisal and procurement is expected to differ across countries. The objective of this research was to review healthcare sustainability policy and implementation progress across the EU5.

**Methods:**

Healthcare sustainability policies in the EU5 (France, Germany, Italy, Spain, and the UK) were reviewed. Policies were identified via government websites (e.g., the Ministere de la sante et de la prevention) and through review of climate and sustainability policy news on the WHO website. Common goals featured in the policies included carbon reduction plans, reduction of single use plastics, ethical and environmental considerations, and self-sustainability. These four goals were selected to form sustainability categories for comparisons across markets. The policies were reviewed for each of the categories with respect to level of inclusion, targets, and anticipated implementation dates.

**Results:**

The UK had clear policies focusing on carbon and emission reduction. Implementation of these policies began in April 2023 and will become a requirement for all new procurements from April 2024. France, Italy, and Spain mentioned carbon emission reduction and environmental impact in policies, but targets and guidelines have yet to be set. Italy and the UK had policies relating to reduction of single-use plastics, whereas the other markets did not. Germany had limited policies mandating changes geared toward increased environmental sustainability, focusing instead on self-sustainability and resilience in the market.

**Conclusions:**

As expected, incorporation of sustainability plans into healthcare policy is variable. Within markets with established policies, such as the UK, there is progress toward mandating the inclusion of sustainability policy in procurement, the impact of which may be seen in early 2024. Although the progress of implementing sustainability in other markets is slower, countries are moving toward environmentally sustainable healthcare policies.

